# Influence of Fabrication Techniques on the Physicochemical, Textural, Release and Skin Delivery Performance of Polyvinyl Alcohol-Based Transdermal Films Containing Menthol: A Comparative Study

**DOI:** 10.3390/pharmaceutics18070871

**Published:** 2026-07-16

**Authors:** Gintaras Matulis, Yuliia Maslii, Nataliia Herbina, Mindaugas Marksa, Agnė Mazurkevičiūtė, Jurga Bernatoniene

**Affiliations:** 1 Department of Drug Technology and Social Pharmacy, Lithuanian University of Health Sciences, LT-50161 Kaunas, Lithuania; gintaras.matulis@lsmu.lt (G.M.); yuliia.maslii@lsmu.lt (Y.M.); nataliia.herbina@lsmu.lt (N.H.); 2 Department of Analytical and Toxicological Chemistry, Lithuanian University of Health Sciences, LT-50161 Kaunas, Lithuania; mindaugas.marksa@lsmu.lt; 3 Institute of Pharmaceutical Technologies, Faculty of Pharmacy, Medical Academy, Lithuanian University of Health Sciences, LT-50161 Kaunas, Lithuania; agne.mazurkeviciute@lsmu.lt; 4 Department of Clinical Pharmacy, Lithuanian University of Health Sciences, LT-50161 Kaunas, Lithuania

**Keywords:** transdermal films, polyvinyl alcohol, menthol, 3D printing, solvent casting, electrospinning, drug release, skin permeation

## Abstract

**Background:** Transdermal films are promising dosage forms for controlled delivery of active pharmaceutical ingredients through the skin. Polyvinyl alcohol (PVA)-based matrices are particularly attractive due to their biocompatibility, film-forming ability, and versatility. However, the development of films for volatile compounds such as menthol remains challenging due to high potential losses during processing and application. This study compares PVA-based transdermal films fabricated by 3D printing, solvent casting, and electrospinning, focusing on the effect of fabrication method on film properties, release behaviour, and skin delivery performance. **Methods:** A comprehensive characterization included morphology, structure, thickness, moisture content, mechanical properties, adhesion, menthol content, in vitro release, ex vivo permeation, and stability. **Results:** Fabrication method significantly influenced film microstructure, menthol entrapment, and stability. Menthol acted as a plasticiser, increasing thickness and moisture content while reducing mechanical strength via disruption of intermolecular interactions within the PVA matrix. The 3D-printed films exhibited the highest entrapment efficiency (14.40%, corresponding to 4.00% menthol content in the dried matrix) and superior menthol retention after 6 months (75.0%), compared to solvent-cast and electrospun films, due to their dense layered structure limiting volatile losses. All formulations showed biphasic release behaviour, strongly dependent on fabrication method. Electrospun films released menthol fastest (68.94% at 1 h), followed by solvent-cast films (63.48% at 1 h), whereas 3D-printed films exhibited a more sustained profile (46.14% at 2 h), reflecting differences in porosity and diffusion pathways. These structural differences also affected skin delivery, with 3D-printed systems demonstrating higher epidermal flux than the other formulations. **Conclusions:** Overall, fabrication method governed film microstructure and thereby controlled menthol entrapment, release, and transdermal performance. Extrusion-based 3D printing offers a promising strategy for designing transdermal systems for volatile compounds with improved structural control and delivery efficiency.

## 1. Introduction

Transdermal delivery of analgesic agents is currently considered a promising approach for treating pain syndromes of various etiologies. This method of administration enables localized or systemic analgesic effects while minimizing systemic exposure. Transdermal dosage forms bypass the gastrointestinal tract and first-pass metabolism in the liver. This enhances the bioavailability of active substances and reduces the risk of side effects [1,2]. Particular attention is given to locally acting analgesic agents, among which menthol is included.

Menthol is one of the most widely used ingredients in topical pharmaceutical formulations due to its pronounced cooling, anti-itching, and pain-relieving effects [3,4,5]. It is commonly included in products intended for the symptomatic treatment of pain, inflammation, and itchiness of the skin and underlying tissues.

The pharmacological activity of menthol is primarily associated with the activation of cold-sensitive transient receptor potential melastatin 8 (TRPM8) channels located on peripheral sensory nerve endings in the skin [6,7,8]. Stimulation of these receptors induces a cooling sensation, producing an analgesic and antipruritic effect through sensory modulation and distraction. Unlike local anesthetics, menthol does not block nerve conduction but rather alters sensory perception. This contributes to its favourable safety profile and good tolerability when applied topically.

Various formulation developments incorporating menthol as an active pharmaceutical ingredient (API) have been reported in the literature, with most studies focusing on semisolid dosage forms such as gels, creams and emulsions [5,9]. It has been demonstrated that menthol can enhance skin permeability and facilitate the transdermal delivery of active substances within such systems. In particular, combined gel and nanoparticle-based formulations containing menthol have been shown to improve skin penetration and provide more pronounced therapeutic effects than menthol-free systems [10,11,12].

However, the development of medicated polymeric films incorporating menthol and data on its skin penetration from such film-based delivery systems are largely absent from the available literature, indicating a significant research gap in this area. Incorporating this agent into transdermal systems may contribute to achieving a prolonged analgesic effect and improving therapeutic efficacy.

Polymeric drug films are widely used to take advantage of transdermal delivery of analgesic agents. These thin, flexible systems have active substances uniformly distributed within a polymer matrix [13,14]. Using polymeric films enables the controlled release of active components, precise dosing, and protection of drugs from external factors. A key advantage of these systems is the ability to modify the composition of the film-forming polymers and adjust their physicochemical and mechanical properties. These properties influence skin adhesion, drug release rate, and bioavailability [15].

Polymeric drug films can be produced using various methods, including solvent casting, melt casting, extrusion, electrospinning, etc. [16,17,18,19]. Recently, there has been a particular focus on using additive technologies, especially three-dimensional (3D) printing, to fabricate film-based dosage forms. Three-dimensional printing enables precise control over film thickness, structure, and composition. This ensures the uniform distribution of active substances and the reproducibility of the final product’s characteristics. Additional advantages of 3D-printed polymeric films include dose personalization, creation of multicomponent systems, and adaptation of the dosage form to individual patient needs, making this approach particularly promising for developing modern transdermal analgesic formulations [20,21,22].

Polyvinyl alcohol (PVA) is widely used in developing transdermal systems because of its combination of safety, biocompatibility, biodegradability, excellent film-forming properties, and high compatibility with various pharmaceutical agents [23,24,25]. PVA’s amphiphilic nature, resulting from the presence of hydrophilic hydroxyl groups and a hydrophobic carbon backbone, allows for the efficient incorporation of drugs with diverse physicochemical properties [26,27]. Studies on PVA-based hydrogels further support the potential of PVA, demonstrating controlled drug release, good skin adhesion, and applicability in wound dressings and transdermal patches [28,29].

From a technological perspective, a key advantage of PVA is the relatively low and easily adjustable viscosity of its aqueous solutions, which allows the production of film-based dosage forms using various methods, including solvent casting, electrospinning, and additive technologies such as 3D printing. The good flow properties and homogeneity of PVA solutions contribute to the stability of the film-forming process, uniform distribution of active substances within the polymer matrix, and reduced risk of nozzle clogging in processing equipment. The ability of PVA to form strong, elastic, and transparent films, combined with its technological versatility, makes this polymer one of the most preferred materials for the development of transdermal drug delivery systems [25,30,31,32].

Despite the widespread use of 3D printing and conventional film-forming methods in pharmaceutical practice, systematic studies comparing transdermal delivery methods of analgesics, particular menthol, remain limited. This study aims to address this gap by using comparable polymer matrix compositions and drug loadings across all methods. This allows for a direct assessment of how the fabrication technique impacts the films’ technological and biopharmaceutical properties.

This study aims to develop and compare the efficacy of PVA-based transdermal polymeric films that incorporate the analgesic active pharmaceutical ingredient—menthol—using various fabrication techniques. Particular focus is given to investigating how manufacturing methods influence menthol accumulation in the epidermis and the overall efficiency of transdermal drug delivery.

## 2. Materials and Methods

### 2.1. Materials

APIs: Racemic menthol (±-menthol, ≥98.0% by GC, catalog no. 63670-500G-F, lot BCBT6056, Sigma-Aldrich Chemie GmbH, Steinheim, Germany).

Excipients: Polyvinyl alcohol (PVA, MW 30,000–70,000, 87–90% hydrolyzed, Sigma-Aldrich, Steinheim, Germany) was used as the primary film-forming polymer. Glycerol (SOLVAGREEN^®^, ≥86%, Ph. Eur., pharmaceutical grade, Art.-Nr. 7533.3, Carl Roth GmbH + Co. KG, Karlsruhe, Germany) served as plasticizer. Ethanol (96%, pharmaceutical grade, Fabric Vilniaus degtinė, Vilnius, Lithuania) and deionized water (resistivity >18 MΩ·cm, obtained from laboratory purification system GFL Typ 2004, Gesellschaft für Labortechnik mbH, Burgwedel, Germany) were used as solvents.

Analytical Reagents: acetonitrile (HPLC gradient grade, ≥99.9%), formic acid (LC-MS grade, ≥98%), methanol (≥99%, Sigma–Aldrich, Taufkirchen, Germany) and analytical-grade water. All solvents were obtained from commercial suppliers certified for LC-MS applications.

All chemicals were used as received without further purification and stored according to manufacturer recommendations. Chemical identity and purity were verified by supplier certificates of analysis.

### 2.2. Preparation of Film-Forming Solution and Film Fabrication

Three polymeric film formulations were developed using different fabrication strategies: 3D printing, solvent casting, and electrospinning. Their compositions are presented in Table 1.

Polyvinyl alcohol was dissolved in purified water at 80–90 °C with constant stirring, then cooled to room temperature. Glycerol was then added to the prepared aqueous phase while stirring continuously. Separately, menthol was dissolved in ethanol to prepare the alcoholic phase. The aqueous phase was then gradually added to the alcoholic phase under constant stirring to obtain a homogeneous hydroalcoholic solution. The final mixture was left to stand for 3–4 h to allow the air bubbles to escape. This solution was used to prepare films by three different methods. The same amount of starting solution (6.0 g) was used for film formation in all three methods. Each film formulation was prepared in at least three independent batches to ensure the reproducibility of the fabrication process.

#### 2.2.1. Viscosity Measurement of Film-Forming Solution

The viscosity was measured using a Fungilab Alpha series rotational viscometer (Fungilab, Barcelona, Spain). Equal amounts (50.00 ± 0.01 g) of the samples were analysed with an L2 spindle at a shear speed of 50 rpm. Three replicate analyses were performed at room temperature for each sample.

#### 2.2.2. Semi-Solid Extrusion (SSE) 3D Printing Method

Digital film models were prepared in Autodesk Inventor 2026 (Autodesk, Portland, OR, USA) CAD software. Each batch consisted of 2 circle films of 85 × 85 mm base and 0.1 mm height (5 × 20 µm layer) were printed. These designs were saved as a stereolithographic file (.stl) and exported to the PrusaSlicer 2.9.3 (Prusa Research Ltd., Praha, Czech Republic). This study utilized an SSE 3D printer with 2 independent extruders designed as linear syringe pumps. Syringes of 60 mL were used to supply the material through a 50 cm tubing to a nozzle (0.82 mm dia.). Films were printed on 90 µm thick polyester masking tape (Lepíky Ltd., Praha, Czech Republic) laid on a 2 mm glass sheet. The print settings were as follows: bed temperature 35 °C, print speed 15 mm/s, extrusion width 0.82 mm, 1 perimeter, and 100% circle pattern infill density. After finishing the print, two temperature regimes were used to provide sufficient drying of the films: room temperature (25 °C) and bed temperature (35 °C) [33].

#### 2.2.3. Solvent Casting Method

The prepared solution was poured evenly into Petri dishes. To ensure sufficient drying, two temperature regimes were evaluated: room temperature (25 °C) and 35 °C, until a uniform film was obtained that could be easily detached from the substrate. The resulting films were covered with wax paper and stored in desiccators until further evaluation tests were performed [34].

#### 2.2.4. Electrospinning Method

The electrospinning system was constructed using a LA-120 syringe pump (Jiangsu Zhengkang Medical Apparatus, Shanghai-Nanjing Railway, Changzhou, China), a high-voltage power supply (Model HVGD-30, Gamma High Voltage Research Inc., Ormond Beach, FL, USA), a syringe with a needle, and a collector. A voltage of 15 kV was applied, and the electrospun nanofibrous films were collected on a grounded aluminium pan (dia. 85 mm) at a distance of 10 cm from the syringe tip. The electrospinning setup was mounted on a DOBOT MG400 robotic arm (Shenzhen Yuejiang Technology Co., Ltd., Shenzhen, China) equipped with a programmable X–Y positioning stage. The traverse speed of the needle relative to the collector was maintained at 50 mm/s to ensure uniform fiber deposition. The electrospinning process was carried out for 1 h at a temperature of 25 °C and a relative humidity of approximately 40–50% [35].

**Figure 1:**
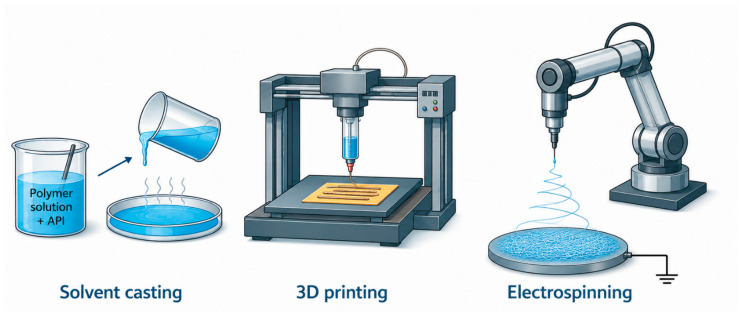
An illustration of the film production process.

A schematic representation of the film fabrication process is shown in Figure 1.

### 2.3. Film Characterization

#### 2.3.1. Organoleptic Evaluation

Parameters such as color, transparency, surface smoothness, uniformity, flexibility, and ease of handling were visually and manually inspected. Any presence of cracks, bubbles, or uneven thickness was noted. Odor and general appearance were also assessed to ensure consistency and suitability for topical application.

#### 2.3.2. Scanning Electron Microscopy (SEM) Analysis of Film Surfaces

The samples were put on the aluminum holder. They were observed under a scanning electron microscope Hitachi S-3400N (Hitachi High Technologies, Minato, Tokyo, Japan) at a magnification of 1000× (at 5.00 kV, SE detector, and the working distance of 8 mm).

#### 2.3.3. X-Ray Diffraction (XRD) Analysis

XRD measurements were performed using a D8 Discover X-ray diffractometer (Bruker AXS GmbH, Karlsruhe, Germany) equipped with a Cu K_α_ radiation source (λ = 1.54 Å). The measurements were conducted in parallel beam geometry using a 60 mm Göbel mirror. A Soller slit with an axial divergence of 2.5° and a 1.2 mm divergence slit were employed on the primary beam path. On the secondary side, a LYNXEYE position-sensitive detector (Bruker AXS GmbH, Karlsruhe, Germany) operating in 1D mode was used, with an opening angle of 2.16° and a slit width of 2.0 mm. The X-ray generator was operated at 40 kV and 40 mA. Diffraction patterns were recorded over the 2θ range of 5–90° with a step size of 0.043° and a dwell time of 19.2 s per step, using an automatic repeat function. Data processing and analysis were performed using DIFFRAC.EVA software v3.0 (Bruker AXS GmbH, Karlsruhe, Germany).

#### 2.3.4. Attenuated Total Reflectance Fourier Transform Infrared (ATR-FTIR) Analysis

ATR-FTIR spectra were recorded using a Nicolet 6700 FTIR spectrometer (Thermo Fisher Scientific, Waltham, MA, USA) equipped with a diamond attenuated total reflectance (ATR) crystal and a deuterated triglycine sulfate (DTGS) detector. Background spectra were collected before each measurement. Spectra were acquired over the range of 4000–400 cm^−1^ using 32 scans at a spectral resolution of 4 cm^−1^. Each sample was measured in triplicate, and the obtained spectra were averaged prior to analysis.

#### 2.3.5. Thickness Measurement

The thickness of the films was measured at five different locations using an Electronic Digital Caliper DIN 862 (Vogel Germany GmbH & Co. KG, Kevelaer, Germany) and an average value was calculated. Three films of each composition were taken for the study.

#### 2.3.6. Moisture Measurement

Moisture was measured with Moisture analyzer KERN DBS60-3 (KERN & SOHN GmbH, Balingen, Germany). A sample (0.1 ± 0.05 g) was weighed and heated (105 °C) to a constant mass, and moisture content (%) was shown in a device’s screen. The weight loss observed during this process, quantified as a percentage, accurately reflects the product’s moisture content.

#### 2.3.7. Folding Endurance Test

This value is used to evaluate the flexibility of the polymeric film. To determine folding endurance, three samples from each formulation were cut into 20 × 20 mm squares. Each film was repeatedly folded at the same location until it either broke or reached 300 folds. The number of folds the sample could withstand before breaking, or up 300 times without breaking, was recorded as the folding endurance [36,37,38].

#### 2.3.8. Textural Analysis

Parameters were examined using a TA.XT.plus texture analyzer (Stable Micro Systems Ltd., Godalming, Surrey, UK).

##### The Bursting Strength and Tensile Capacity Test

The probe height calibration was performed prior to the start of the experiment to ensure accurate measurements. The calibration process included the following steps: select T.A. > Calibrate > Calibrate Height from the toolbar menu; set the return distance and speed according to the required parameters, defining how the probe would move after contacting the surface; upon successful probe height calibration, a confirmation window was displayed indicating that the calibration process was completed correctly.

The 3 × 3 cm^2^ film sample was placed between two metal plates with a circular cavity of 1 cm diameter in such a way that no wrinkles or unstressed areas were visible. In this test, a metal probe with a ball at the end (The Film Support Rig HDP/FSR) attached to a metal support was used to pierce the film. The test parameters were as follows: pre-test speed 1.00 mm/s; test speed 5.00 mm/s; post-test speed 10 mm/s; distance 40 mm. During the test, the maximum breaking force (Bursting strength, N) of the sample was recorded and the tensile capacity (Tensile distance, mm), which characterizes the firmness and elasticity of the film, was measured.

##### The Tensile (Elongation) Test

The analyzed films cut to a size of 6 × 1 cm were attached from both ends to clamps on opposite sides of the A/TG probe. The clamps were held on a metal base. One of the clamps was fixed and the other was movable. During the measurement, the moving clamp pulled the film upward. The stretching speed was 6 mm/s and the lifting distance of the moving clamp was 100 mm. Tensile strength (N/mm) was measured when the film was torn, providing an indication of its elasticity and plasticity.

##### Assessment of Adhesion

A completely dry piece of film measuring 2 × 2 cm^2^ was placed on a special fixed plastic table and pressed with a transparent plastic plate with a 1 cm diameter cavity in the middle, leaving an open part of the film (A/MUC adhesive probe). The open part of the film was previously moistened with a small amount (100 μL) distilled water (pH ≈ 5.5–6.5) at room temperature, the hydration time was 15 s. Then the P/0.5R probe with a flat end slightly less than 1 cm in diameter, which was attached to the metal support on top of the texture analyzer, was pressed onto the hydrated film: the force applied during contact—5 N, the contact time—60 s. Then, the force required to detach the probe end from the film surface (adhesion) was measured.

#### 2.3.9. Visual Observation of Film Behavior on the Skin

To evaluate the physical behavior and structural integrity of the formulation matrices upon contact with a moist biological surface, a qualitative in vivo assessment was performed on human skin (the hand). The skin surface was pre-moistened with distilled water to ensure uniform wetness. Dry film samples (3 × 3 cm^2^) were applied to the prepared area. The subsequent swelling, disintegration, and matrix dissolution behaviors of the different formulation batches (F1, F2, and F3) were tracked via continuous visual monitoring and documented photographically. This qualitative assessment was conducted in accordance with the Declaration of Helsinki and approved by the Kaunas Regional Biomedical Research Ethics Committee, Lithuanian University of Health Sciences, Kaunas, Lithuania (Protocol No. BE-2-42). Informed consent for participation was obtained from the volunteer prior to the assessment.

#### 2.3.10. Determination of Menthol Content Using Gas Chromatography

For the extraction of menthol, the film samples (accurate amount) are dissolved in a mixture of water and methanol (5:5) in a volume of 10 mL. The samples were placed in an ultrasonic bath for 10 min, then filtered through a membrane filter. The resulting solution was used for gas chromatography with flame ionization detector (GC/FID) analysis.

GC/FID analysis was performed using a Shimadzu GC-2010 Plus instrument (Shimadzu Technologies, Kyoto, Japan). The analytical conditions were as follows: volume injected: 1 μL; carrier gas: helium at 1.26 mL/min; injector temperature: 260 °C; flame ionization detector temperature: 315 °C; split ratio: 1:60; oven temperature: ranging from 50 to 315 °C with a stepwise temperature programme within the total run time of 23.53 min. A 30 m Restek Rxi-5ms (Restek Corporation, Bellefonte, PA, USA) column with a diameter of 0.25 µm and a thickness of 0.25 µm was used for analysis. For quantitative evaluation, a calibration graph was constructed using menthol standard solutions of different concentrations. The calibration range was 0.00078–0.4 µg/mL; the formula was Y = 169,748x + 68.2398; the R^2^ value was 0.99996; and the retention time of menthol was 7.451 min.

To evaluate the capability of each manufacturing method to retain the volatile API during processing, the menthol entrapment efficiency (%EE) was calculated using the following Equation (1):

(1) $$\%\mathrm{EE}=\frac{C_{\mathrm{practical}}}{C_{\mathrm{theoretical}}}\times 100\%,$$

where C_practical_ is the actual percentage of menthol determined in the dried film matrix via GC/FID, and C_theoretical_ is the theoretical target percentage of menthol calculated based on the initial dry weight of all formulation components (PVA, glycerol, and menthol), which equaled 27.78%.

#### 2.3.11. In Vitro Menthol Release Study

Menthol release from polymeric films was evaluated using a paddle-type dissolution apparatus (USP II) (Sotax AT 7Smart Dissolution system, Sotax AG, Aesch, Switzerland) [39,40]. Film samples were placed in a release medium consisting of phosphate buffer (pH 7.4) and 96% ethanol (70:30, *v/v*) at a film-to-medium ratio of 1:50 (*w/v*). Dissolution vessels were filled with 500 mL of release medium and maintained at 32 ± 0.5 °C under continuous stirring at 50 rpm. To ensure complete immersion and prevent floating, the films were secured to a stainless-steel wire mesh. At predetermined time intervals (0.5, 1, 2, 3, 4, 5, 6, and 24 h), 2 mL aliquots of the release medium were withdrawn and immediately replaced with an equal volume of fresh preheated medium to maintain constant volume and sink conditions. The collected samples were filtered through a 0.2 μm syringe filter and analysed by GC-FID (Shimadzu GC-2010 Plus) as described above. All experiments were performed in triplicate. To account for the highly volatile nature of the active substance and its simultaneous evaporation from the dissolution medium, the release behavior was expressed as the time-dependent profile of the percentage of menthol remaining in the medium relative to its initial content in the matrix, rather than a traditional cumulative release profile.

#### 2.3.12. Ex Vivo Skin Permeation Studies

Ethical Approval and Donor Selection: Human abdominal skin from Caucasian female donors (ages 30–55, *n* = 10) undergoing elective abdominoplasty at Hospital of Lithuanian University of Health Sciences. Protocol approved by Kaunas Regional Biomedical Research Ethics Committee (Lithuanian University of Health Sciences, Kaunas, Lithuania; Protocol No. BE-2-42, approved 2023-05-03) in accordance with Declaration of Helsinki. Informed consent for participation was obtained from all subjects involved in the study. Inclusion criteria: female patients 30–55 years with no chronic skin diseases, diabetes, or systemic corticosteroid use within 3 months [41].

Skin Preparation: Immediately post-excision, subcutaneous fat and fascia carefully removed, skin rinsed with isotonic saline, cut into 0.64 cm^2^ sections, individually wrapped in aluminum foil, frozen at −20 °C (maximum 6 months). Storage validation studies confirmed no significant barrier function changes over 6 months at −20 °C [42,43].

Permeation Study Protocol: Bronaugh-type vertical flow-through diffusion cells (custom glass cells, local manufacturer to published specifications) [42,44,45].

Parameters: diffusion area 0.64 cm^2^ (9 mm diameter circular orifice), receptor chamber volume 7 mL, receptor medium phosphate-buffered saline (PBS, pH 7.4, 0.01 M) with 0.5% Tween 80 (to enhance menthol solubility >12 mg/mL ensuring sink conditions), temperature 32 °C (skin surface temperature, maintained by circulating water jacket), flow rate 1.5 mL/h (peristaltic pump, Watson-Marlow 505S (Watson-Marlow Limited, Falmouth, Cornwall, UK)), study duration 24 h.

Full-thickness skin mounted between donor and receptor chambers with stratum corneum facing upward, secured using silicone O-ring seals preventing edge leakage. After 30-min equilibration, film samples (~0.5 g, providing infinite dose conditions) applied to skin surface. Donor chambers covered with aluminum foil (light protection, minimize evaporation).

Tissue Processing: After 24 h, film residue removed using forceps, skin surface gently rinsed with distilled water (3 × 5 mL) and blotted with filter paper. Epidermis separated from dermis using heat separation technique: skin sample pressed (epidermal side down) against heated stainless steel surface (60 ± 1 °C) for exactly 10 s, then epidermis carefully peeled away [46,47]. Separated epidermis and dermis were placed in separate 2 mL microcentrifuge tubes, each extracted with 1 mL of ethanol (96%), vortexed for 30 s, sonicated for 30 min at room temperature, and centrifuged at 10,000× *g* for 10 min at 4 °C. The resulting supernatants were transferred to GC vials, filtered through a 0.22 μm PTFE membrane, and analyzed by GC–FID as described above.

Heat Separation Validation: Skin samples (*n* = 6) spiked with known menthol concentrations (10, 50, 100 μg/mL) before and after heat separation (60 °C, 60 s). Recovery rates: 97.2–102.8% (RSD < 5%), indicating no significant menthol loss or redistribution. This validates observed tissue distribution reflects true permeation patterns, not processing artifacts [48].

### 2.4. Stability Study

The stability of the developed films was evaluated after 6 months of storage under the specified conditions. Film samples were individually sealed in plastic bags and stored at room temperature (20–25 °C) under ambient relative humidity (30–40% RH). After the storage period, the films were visually inspected for changes in appearance, including colour, transparency, flexibility, surface integrity, and the presence of cracks or deformation. In addition, menthol content was determined by GC-FID (Shimadzu GC-2010 Plus) using the analytical procedure described above. Menthol retention (%) was calculated by comparing the menthol content after 6 months with the initial content measured immediately after film preparation. All measurements were performed in triplicate.

### 2.5. Statistical Analysis

All statistical analyses were performed using GraphPad Prism version 10.4.0 for Windows (GraphPad Software, Boston, MA, USA). Data are presented as mean ± standard deviation (SD). To evaluate the combined influence of fabrication method and formulation factors on the physicochemical and textural characteristics of the films, a two-way analysis of variance (ANOVA) followed by Tukey’s post hoc test was used (*n* = 5). For comparisons where the fabrication method was considered as a single independent variable, a one-way ANOVA followed by Tukey’s multiple comparisons post hoc test was applied (*n* = 3). This approach was used to assess statistically significant differences in time-dependent menthol release profiles and ex vivo skin permeation fluxes. In all cases, differences were considered statistically significant at *p* < 0.05.

## 3. Results and Discussion

In Europe, menthol is widely incorporated into nationally authorized topical medicinal products to provide symptomatic relief for musculoskeletal pain and to modulate local nociceptive responses. For instance, Deep Relief Joint Pain Gel, authorized in the United Kingdom, combines ibuprofen with approximately 3% *w*/*w* menthol to leverage its characteristic cooling and counter-irritant effects. Similarly, high-strength topical analgesics, such as Deep Heat Max Strength cream, utilize menthol at concentrations up to 8% *w*/*w* alongside methyl salicylate to deliver robust rubefacient and analgesic actions. These commercial examples illustrate that a concentration window of 3% to 8% *w*/*w* represents the established European pharmaceutical standard for maintaining an optimal balance between sensory perception, local therapeutic efficacy, and skin tolerance.

Within this clinical and technological framework, a menthol concentration of 5% *w*/*w* was selected for the developed film-forming formulations, aligning with robust literature data on topical and transdermal delivery systems. Specifically, Fang et al. demonstrated that incorporating 5% menthol into a topical gel base provides efficient local analgesia while successfully enhancing the skin permeation of co-administered active substances [49]. In a similar vein, Krishnaiah et al. utilized a 5% menthol loading in a membrane-moderated transdermal system for nicardipine hydrochloride, confirming its potency as a biocompatible penetration enhancer [50]. Furthermore, a comprehensive mechanistic review by Sapra et al. highlighted that within the typical terpene optimization range, a 5% concentration maximizes matrix-to-skin flux while ensuring excellent tissue compatibility and minimizing local irritation [51]. Therefore, the chosen 5% *w*/*w* concentration serves a dual purpose in this study: acting as a clinically relevant active component and functioning as an optimized permeation enhancer.

The development of a film-forming matrix for the co-delivery of hydrophilic and hydrophobic compounds requires a suitable solvent system capable of dissolving both types of components. PVA is a highly hydrophilic polymer and requires water to achieve complete dissolution [52,53,54]. In contrast, menthol, used as a model lipophilic API, exhibits very low solubility in water and therefore requires ethanol as a co-solvent [3,55,56]. To obtain a homogeneous single-phase solution without visible phase separation, a binary water–ethanol solvent system was investigated. The selected PVA grade (87–90% hydrolyzed) is particularly suitable for aqueous-alcoholic formulations because the residual acetate groups improve its compatibility with ethanol-containing media. As a result, this polymer remains soluble in water–ethanol mixtures containing relatively high ethanol fractions (40–50%). To identify the optimal solvent composition, the ethanol-to-water ratio was systematically varied (0.5:1, 1:1, and 1.5:1, *w*/*w*) while maintaining a preliminary PVA concentration of 10% *w*/*w*, selected based on commonly reported film-forming formulations [57,58,59]. The objective was to determine a solvent composition that ensures complete dissolution of 5% menthol while maintaining full PVA solubility and solution homogeneity. The visual characteristics and technological performance of the resulting formulations are summarized in Table 2.

The results presented in Table 2 showed that the stability and appearance of the PVA/menthol system were strongly dependent on the ethanol-to-water ratio. The extreme solvent compositions did not provide satisfactory formulations due to the differing solubility requirements of the hydrophilic polymer and the lipophilic active compound. At the lower ethanol content (0.5:1 *w*/*w*), incomplete menthol solubilization was observed, resulting in visible phase separation. In contrast, at the higher ethanol content (1.5:1 *w*/*w*), rapid polymer aggregation and precipitation occurred, producing a heterogeneous multiphase system. Among the tested formulations, the 1:1 (*w*/*w*) ethanol-to-water ratio provided the most favorable outcome, yielding a homogeneous, single-phase opalescent solution with no visible signs of separation or precipitation during evaluation. Based on these observations, the 1:1 ethanol-to-water ratio was selected as the optimal solvent system and used in all subsequent experiments.

Following the selection of the solvent system, the concentration of the film-forming polymer was optimized. PVA is commonly used in pharmaceutical and cosmetic film formulations at concentrations ranging from 5% to 15% *w*/*w* [60,61,62]. Within this concentration range, polymer content strongly influences the viscosity, handling properties, and film-forming performance of the solution [30,63,64]. Therefore, a series of formulations containing different PVA concentrations was prepared and evaluated to identify a composition that provided suitable processing characteristics while maintaining formulation homogeneity and stability.

Since the developed formulation was intended for three different manufacturing approaches—SSE 3D printing, solvent casting, and electrospinning—the concentration of PVA was systematically optimized. Polymer concentrations of 5%, 10%, and 15% *w*/*w* were evaluated to determine a composition suitable for all processing methods. Polymer concentration strongly influences the viscosity and handling characteristics of the formulation. Solutions with insufficient polymer content generally exhibit poor structural stability, whereas excessively concentrated systems may become difficult to process. Therefore, the selected formulation had to provide adequate flow properties during casting and electrospinning while maintaining sufficient consistency for stable extrusion during 3D printing.

To improve the flexibility of the resulting matrices, glycerin was incorporated as a biocompatible plasticizer. Glycerin interacts with the PVA network through hydrogen bonding, increasing chain mobility and reducing intermolecular interactions, which ultimately improves the flexibility and handling properties of the dried films [65,66]. The concentration of glycerin was systematically optimized to achieve an appropriate balance between flexibility and structural integrity. Preliminary screening demonstrated that a low glycerin content (1% *w*/*w*) was insufficient plasticization capacity, resulting in rigid and brittle matrices that frequently fractured during peeling and routine handling. In contrast, increasing the glycerin concentration to 5% *w*/*w* and above resulted in excessive plasticization. These formulations developed a greasy and highly tacky surface, indicating migration of excess glycerin to the film surface during drying. As a consequence, the resulting matrices exhibited poor mechanical stability, increased stickiness, and a tendency to deform during handling. Similar behavior was also observed during SSE 3D printing and electrospinning, where excessive tackiness promoted filament coalescence and compromised shape retention. Based on these observations, 3% *w*/*w* glycerin was selected as the optimal concentration, providing improved flexibility and elasticity while maintaining satisfactory structural integrity, surface characteristics, and processability. The final formulation was adjusted to correspond to an ethanol-to-water ratio of approximately 1:1 (*v*/*v*), expressed in mass fractions for practical preparation. This resulted in a system containing 42% *w*/*w* water and 40% *w*/*w* ethanol, along with menthol (5% *w*/*w*), PVA (10% *w*/*w*), and glycerol (3% *w*/*w*). The formulation remained homogeneous and visually stable, with no phase separation observed.

The technological performance of the investigated formulations and the influence of PVA concentration on processing behavior across the three fabrication methods are summarized in Table 3.

Based on the comparative evaluation of the film-forming matrices (Table 3), a clear correlation was observed between PVA concentration and the rheological behavior of the system, which in turn governed processability across all three fabrication techniques. The 5% *w*/*w* PVA solution exhibited low viscosity (32.41 ± 1.15 mPa·s), corresponding to a dilute, weakly entangled system with limited viscoelastic character. This resulted in insufficient resistance to deformation during processing. In electrospinning, this behavior led to jet instability and bead formation due to electrospraying instead of fiber formation, while in SSE 3D printing it caused loss of shape fidelity and structural collapse of the deposited material. In contrast, the 15% *w*/*w* PVA formulation showed highly viscous, gel-like behavior (745.20 ± 8.63 mPa·s), indicating a densely entangled polymer network. This significantly reduced flowability, leading to restricted mass transfer, slower relaxation of the system, and operational issues such as premature drying and nozzle clogging in both SSE and electrospinning processes. The 10% *w*/*w* PVA solution, with intermediate viscosity (159.03 ± 0.72 mPa·s), provided the most balanced performance. This formulation ensured sufficient fluidity for uniform spreading and deaeration during solvent casting, while maintaining adequate viscoelasticity for stable extrusion in SSE and continuous fiber formation in electrospinning.

Overall, this composition was selected as the optimal formulation for further film fabrication and characterization.

**Figure 2:**
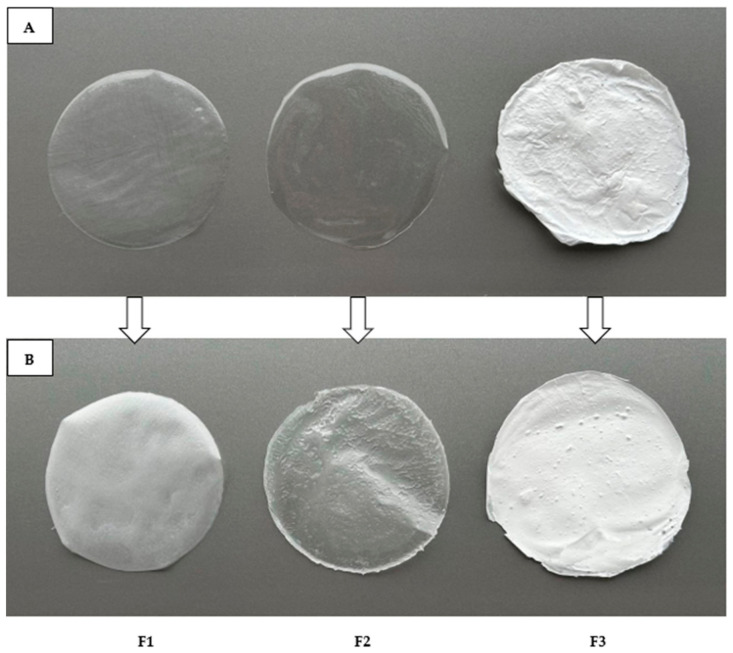
Macroscopic assessment of the appearance of PVA-based films fabricated using different methods: (**A**)—without menthol; (**B**)—with menthol; F1—3D printing; F2—solvent casting; F3—electrospinning.

To obtain PVA-based polymer films measuring 85 mm × 85 mm, three different manufacturing methods were used with the same composition and amount—6.0 g. When films were formed by solvent casting and 3D printing methods without temperature drying, a pronounced tendency of menthol to diffuse into the surface layers of the polymer matrix was observed, which is associated with its limited compatibility with the hydrophilic PVA base and high crystallisation ability [67,68]. Local oversaturation with menthol resulted in phase separation and the formation of white, crystalline inclusions on the surface of the films, that was accompanied by a decrease in their mechanical integrity. Drying at a moderate temperature (35 °C) reduced menthol diffusion migration time and contributed to its more uniform fixation in the polymer matrix, preventing the formation of surface crystalline defects. These phenomena were not observed using the electrospinning, which involves forming the film by applying successive thin layers that dry rapidly at room temperature. The appearance of films with and without menthol is shown in Figure 2.

PVA-based films without APIs (see Figure 2A) produced using the 3D printing (F1) and solvent casting (F2) methods were transparent, homogeneous and elastic. In contrast, the film obtained using an electrospinning method (F3) was white with an uneven surface. The introduction of menthol altered the appearance of the films, except for the sample produced by the electrospinning (F3) (Figure 2B). For the film obtained by solvent casting (F2), a decrease in transparency was observed, as well as the formation of an uneven texture and the appearance of surface inclusions. The film produced using 3D printing (F1) lost its transparency and acquired a white tint; however, it retained its plasticity and smooth surface, demonstrating the best quality of all the menthol samples.

**Figure 3:**
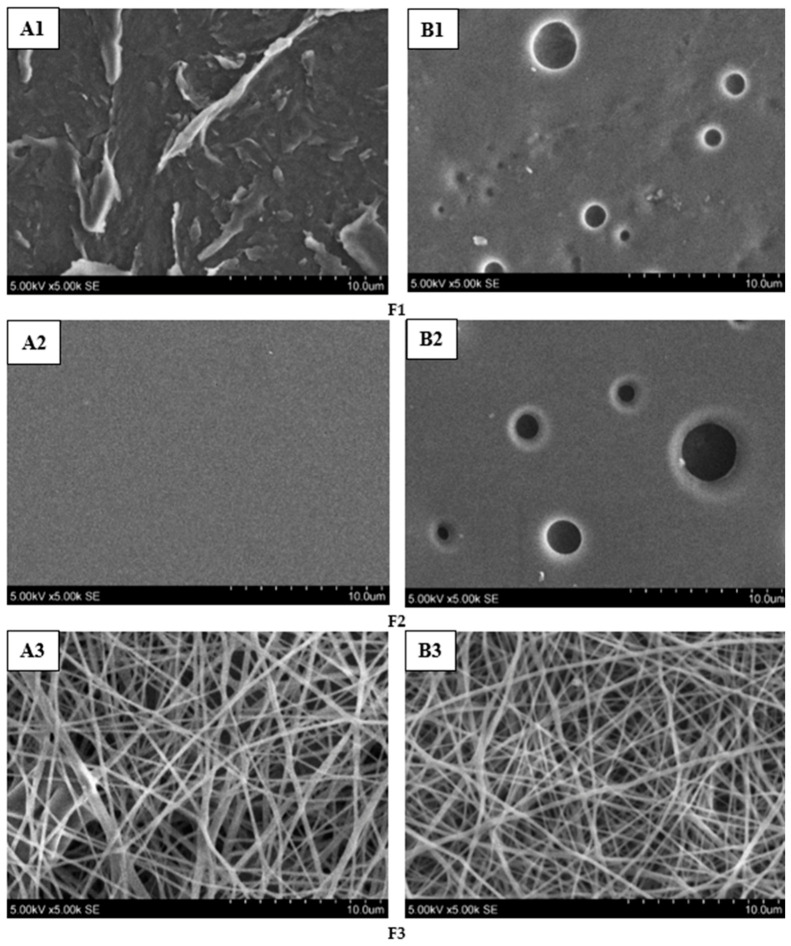
SEM analysis of the micromorphology of PVA-based films fabricated using different methods: (**A1**–**A3**)—without menthol; (**B1**–**B3**)—with menthol; F1—3D printing; F2—solvent casting; F3—electrospinning.

In addition to a macroscopic evaluation of the films’ appearance, their surfaces were examined using SEM at 5000× magnification. This revealed the films’ structural features, which are indistinguishable by visual observation. The results of this analysis are shown in Figure 3.

As shown in Figure 3, SEM analysis revealed distinct morphological differences between the manufacturing methods and drying conditions, as well as the effect of menthol incorporation on the microstructure of the films. The 3D-printed films without menthol (F1–A) exhibited a characteristic microtopography consisting of intersecting lines of deposited material, which is associated with the layer-by-layer deposition process and subsequent solvent evaporation and solidification. The addition of menthol altered the surface morphology of the films (F1–B). When magnified 5000 times, numerous spherical surface depressions, each several micrometres in size, were visible on the films that are probably due to the limited compatibility of menthol with the hydrophilic PVA matrix and its volatility. At elevated drying temperatures, menthol may evaporate or migrate from the surface layers, creating microvoids in the film structure. Furthermore, thermal exposure could intensify the phase separation of the components and disrupt the PVA–glycerol hydrogen bond system.

Films obtained by the solvent casting method without an API (F2–A) at 5000-fold magnification showed a dense, continuous surface due to the uniform distribution of the solution during formation and the formation of a continuous polymer layer. This homogeneous morphology is the result of slow, equilibrium evaporation of the solvent, which allows the polymer chains to relax completely and form a dense matrix. However, films containing menthol (F2–B) showed a significant change in surface morphology: developed porosity was observed, with spherical pores that were deeper and highly heterogeneous in size: these ranged from small individual pores (<2 μm) to larger merged structures (>10 μm). This contrasted with the morphology of films obtained by 3D printing.

The identified differences can be explained by the fact that 3D printing probably ensures a more uniform distribution of menthol throughout the material, as well as a faster formation of the polymer matrix. This reduces the likelihood of large morphological defects being associated with its evaporation.

SEM images of menthol-free films obtained using the electrospinning method (F3–A) showed dense networks of randomly oriented nanofibres. This morphology is characteristic of electrohydrodynamic processing, where the polymer jet is stretched under an applied electric field and rapidly solidifies due to fast solvent evaporation at room temperature, forming a continuous fibrous network. Incorporation of menthol (F3–B) preserved the overall fibrous structure but caused minor morphological changes to the film: only a slight decrease in inter-fibre porosity was observed, accompanied by moderate thickening of the fibres themselves. The lack of heating likely prevented the intense evaporation of menthol and phase separation, as a result of which the mesh structure remained unchanged, suggesting that menthol was incorporated into the nanofibre matrix rather than forming separate phases.

Thus, the SEM results analysis confirms the data obtained from the macroscopic observations: changes in appearance and microstructure were observed in the films produced using solvent casting and 3D printing methods, while no noticeable changes were observed in the films produced using the electrospinning.

**Figure 4:**
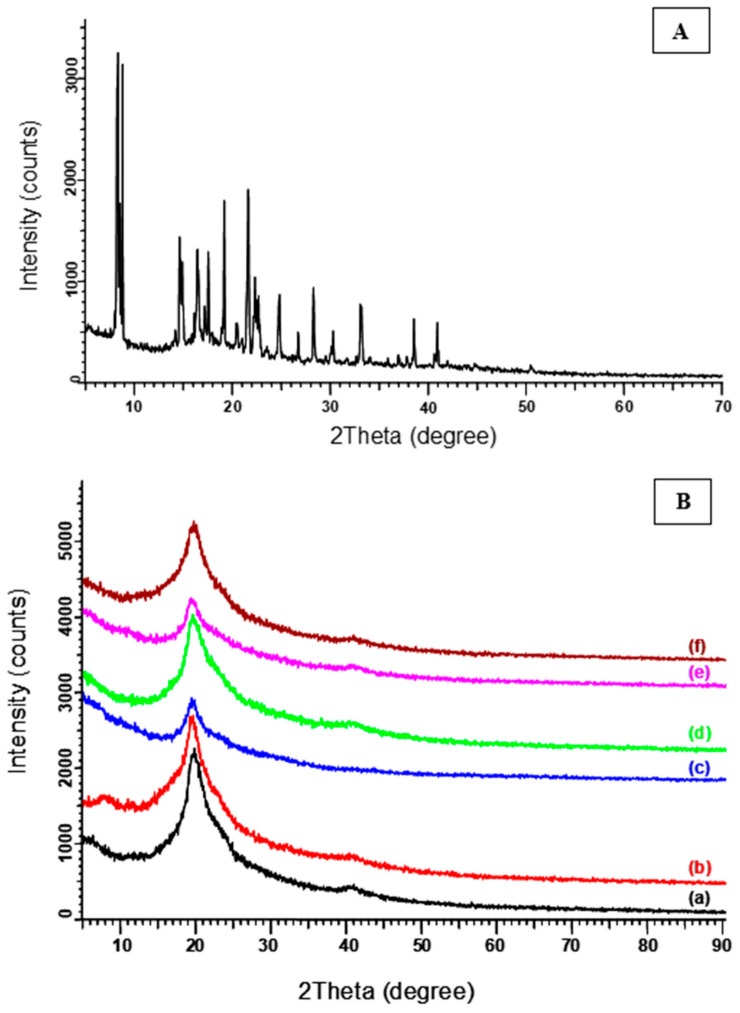
XRD patterns of (**A**) menthol and (**B**) blank and menthol-loaded PVA films fabricated using different methods: 3D printing (a: blank, b: menthol-loaded), electrospinning (c: blank, d: menthol-loaded), and solvent casting (e: blank, f: menthol-loaded).

To further confirm and complement the SEM observations, as well as to gain deeper insight into the internal structural organization of the films, XRD analysis was performed. This technique was used to evaluate possible changes in crystallinity and phase state that may not be directly visible in the surface morphology. The corresponding XRD patterns of pure menthol and the blank and menthol-loaded PVA films prepared by different methods are shown in Figure 4.

The XRD patterns provide insight into the phase state of menthol and the structural organization of the PVA-based films (Figure 4). The diffraction pattern of menthol exhibits numerous sharp and well-defined reflections of varying intensity, confirming its highly crystalline nature. The most intense peaks are observed at low diffraction angles (2θ ≈ 7–9°), where two prominent maxima appear. In addition, several intense reflections are detected in the 2θ range of approximately 14–24°, with maxima around 14°, 16°, 19°, and 22°. Further weaker but well-resolved peaks are present between 25° and 41°, after which the diffraction intensity gradually decreases toward the baseline. The presence of multiple sharp reflections across the entire angular range is characteristic of a well-ordered crystalline lattice and confirms the crystalline structure of menthol. These features are in agreement with previously reported diffraction patterns of this compound [69]. In contrast, all PVA-based films, both blank and menthol-loaded, display a dominant broad diffraction feature centered at approximately 19.5° 2θ. Located within the characteristic diffraction region of semicrystalline PVA [70,71], this feature exhibits pronounced broadening, indicating that the polymer matrix is predominantly amorphous or possesses a low degree of crystallinity. This reduced degree of crystallinity can be attributed to the processing conditions employed during film preparation, which limit the regular arrangement of PVA chains [72]. The effect is particularly pronounced in electrospun films, where ultrafast solvent evaporation kinetically traps the polymer chains before crystalline domains can develop [72,73].

Importantly, no characteristic diffraction peaks of crystalline menthol are detected in any of the menthol-loaded films. The complete disappearance of the characteristic diffraction peaks of menthol indicates the loss of its crystalline structure following incorporation into the PVA matrix. This observation suggests that menthol is present in a molecularly dispersed or amorphous state within the polymer network rather than as a separate crystalline phase, consistent with previous observations for menthol-containing solid dispersions [69] and with the established interpretation of XRD data for amorphous pharmaceutical systems [74]. Furthermore, the diffraction patterns of the blank and menthol-loaded films are nearly identical, demonstrating that menthol incorporation neither alters the supramolecular organization of the PVA matrix nor promotes polymer crystallization.

Overall, the XRD analysis confirms excellent compatibility between menthol and the PVA matrix, resulting in homogeneous films in which menthol is stabilized in a non-crystalline, molecularly dispersed state regardless of the fabrication technique.

**Figure 5:**
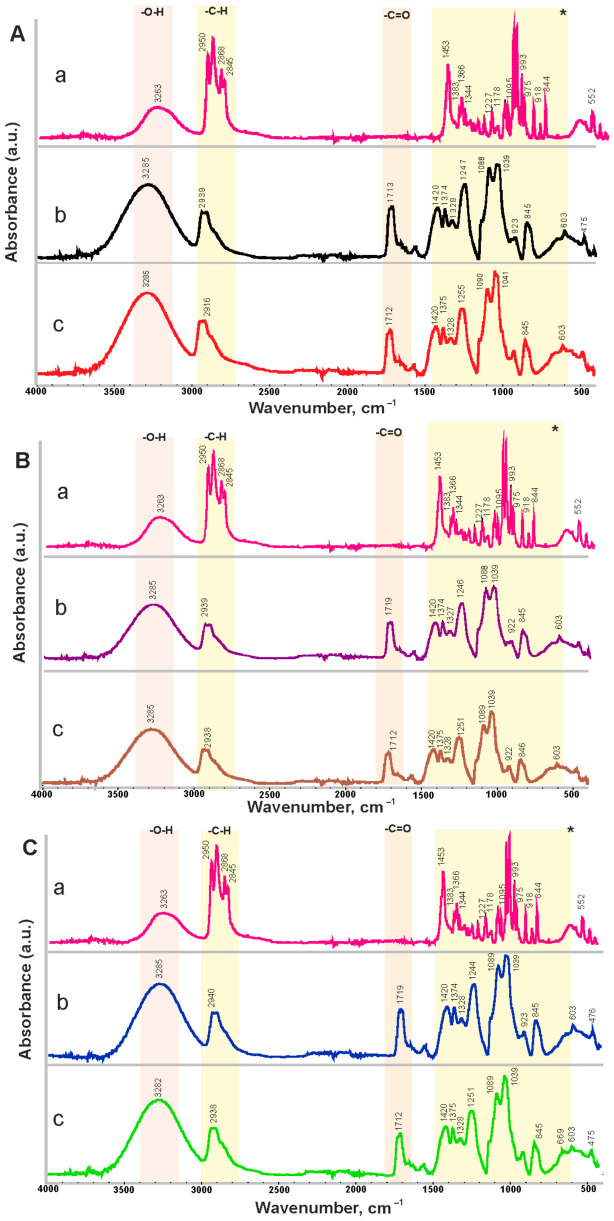
ATR-FTIR spectra of menthol, blank and menthol-loaded PVA films fabricated by different methods: 3D printing (**A**), solvent casting (**B**), and electrospinning (**C**). Sub-panels (**a**–**c**) correspond to menthol, blank, and menthol-loaded samples, respectively; * denotes the expanded fingerprint region.

To complement the morphological (SEM) and structural (XRD) analyses, ATR-FTIR spectroscopy was performed to investigate the interactions between menthol and the PVA matrix and to confirm the successful incorporation of menthol into the film structure. Figure 5 presents the ATR-FTIR spectra of pure menthol and blank and menthol-loaded formulations prepared by 3D printing, solvent casting, and electrospinning. The spectra of the blank formulations are dominated by the characteristic absorption bands of the PVA matrix, whereas the menthol-loaded samples exhibit spectral features attributable to both PVA and menthol, confirming the successful incorporation of the active compound into the films.

The ATR-FTIR spectrum of pure menthol (Figure 5(Aa,Ba,Ca)) exhibits the characteristic spectral features of crystalline menthol. A broad absorption band centered at approximately 3263 cm^−1^ is assigned to O–H stretching vibrations associated with intermolecular hydrogen bonding. Intense bands at 2950, 2868 and 2845 cm^−1^ correspond to the stretching vibrations of aliphatic C–H groups. In the fingerprint region (1500–800 cm^−1^), numerous sharp and well-resolved absorption bands are observed, including signals at approximately 1453–1366 cm^−1^ attributed to C–H bending vibrations, bands in the 1240–1180 cm^−1^ region mainly associated with C–O stretching vibrations, and absorptions below 1000 cm^−1^ corresponding to skeletal vibrations characteristic of the cyclohexane ring. These band assignments are in good agreement with previously reported ATR-FTIR spectra of crystalline menthol [69,75].

The blank PVA films (Figure 5(Ab,Bb,Cb)) exhibit the characteristic ATR-FTIR profile of the polymer regardless of the fabrication method. A broad absorption band centered at approximately 3280–3300 cm^−1^ is assigned to O–H stretching vibrations associated with the extensive hydrogen-bonding network of PVA, whereas the band at approximately 2930–2940 cm^−1^ corresponds to aliphatic C–H stretching vibrations. All blank films display intense absorption in the fingerprint region (1200–900 cm^−1^), corresponding to the characteristic vibrations of the PVA backbone, consistent with previous reports on the structural characterization of PVA. In addition, all blank films exhibit a weak absorption band at approximately 1713–1719 cm^−1^, assigned to C=O stretching vibrations and commonly attributed to residual acetate groups or minor amounts of carbonyl functionalities in PVA, as previously reported in the literature [76,77,78].

In comparison, the menthol-loaded films (Figure 5(Ac,Bc,Cc)) retain the characteristic ATR-FTIR profile of the corresponding blank PVA matrices while exhibiting additional spectral contributions from menthol. The O–H stretching band (3280–3300 cm^−1^) and the aliphatic C–H stretching region (2930–2850 cm^−1^) remain present in all formulations, showing only slight variations in band intensity and position compared with the blank films. These minor spectral changes are consistent with intermolecular interactions between menthol and the polymer chains, most likely involving hydrogen bonding between the hydroxyl groups of PVA and menthol.

No new characteristic absorption bands are observed in the menthol-loaded films, regardless of the fabrication method, indicating that menthol is incorporated into the PVA matrices through physical interactions rather than the formation of new covalent bonds. Minor variations in band intensity and spectral profile among the three fabrication methods may reflect differences in the structural organization of the polymer matrices and the distribution of menthol resulting from the respective processing techniques.

Together with the XRD results, these findings confirm the successful incorporation of menthol into the PVA matrices while preserving the chemical structures of both components. The observed spectral features are consistent with previous reports on PVA-based polymer–drug systems, in which drug incorporation occurs predominantly through non-covalent interactions, particularly hydrogen bonding, without the formation of new chemical bonds [30,79,80].

The structural (XRD) and morphological (SEM) characteristics of the films are expected to govern their physicochemical and mechanical properties. In particular, differences in molecular ordering and the presence or absence of residual crystalline domains may influence polymer chain packing, free volume, and matrix compaction during film formation, thereby affecting film thickness, moisture content, and mechanical behaviour. A comparative analysis of the physicochemical and textural properties was further performed to evaluate the effect of menthol on the film characteristics, as presented in Table 4.

According to the results obtained, the thickness of PVA-based films without menthol significantly depended on the forming method (ANOVA, *p* < 0.05). The minimum thickness was characteristic of the formulation obtained using the solvent casting method (155 ± 9 μm), which involves uniform spreading of the solution over the substrate and effective shrinkage during drying. Samples formed using the 3D printing method were slightly thicker (167 ± 13 μm) due to the layer-by-layer nature of extrusion and the material’s limited self-levelling ability. The greatest thickness was observed in films produced by electrospinning forming (180 ± 12 μm), which was statistically significant compared to the solvent casting method (Tukey’s test, *p* < 0.05). This can be attributed to the lower spreadability of the solution and the formation of a thicker layer during metered spraying.

As a low-molecular-weight hydrophobic compound, menthol acts as a plasticising and structure-modifying additive [3,81,82,83]. Following its introduction, differences in film thickness depending on the method remained, but the absolute values and ratios altered. The thickness of the films produced by 3D printing remained virtually unchanged at 174 ± 14 μm, which indicates that printing parameters play a dominant role in shaping the geometry of the samples. Meanwhile, samples obtained using the solvent casting method exhibited a significant increase in thickness, reaching 171 ± 15 μm (Tukey’s test, *p* < 0.05), which is comparable to the thickness of samples produced by 3D printing. The greatest increase in thickness was observed again for films formed by electrospinning (203 ± 18 µm), demonstrating a statistically significant difference from its blank counterpart (*p* < 0.05) and indicating the method’s high sensitivity to changes in the composition and rheological properties of the film-forming solution.

In general, introducing menthol increased the thickness of PVA-based films for all forming methods, but the extent of this increase depended on the specific features of the technology. The plasticising effect of menthol weakens the intermolecular interactions in the PVA matrix. This leads to a change in the organisation of polymer chains during drying, reducing their degree of compaction. As a result, shrinkage is reduced and a less compact film structure is formed. At the same time, the most pronounced effect was observed in methods dependent on the viscosity of the solution and its spreadability. In 3D printing, however, the effect of menthol is weaker due to the high level of control over the geometry of the layer and the dosage of the material, which cancels out the effect of compositional changes [84,85]. However, since polymeric films are typically defined as thin, continuous materials with a thickness of up to 200 µm [34,86], all samples were practically within the acceptable range, with the electrospun matrix staying at the upper limit of this threshold.

The moisture content of the developed film systems was also evaluated. Precise control of residual moisture is critical, as excess water can alter the films’ mechanical properties, reduce the stability of the API, and accelerate the degradation of the polymer matrix, whereas insufficient moisture adversely affects film flexibility and adhesion [87]. However, given the volatile nature of menthol, it should be noted that the mass loss recorded during the moisture determination procedure may include a minor fraction of co-evaporated active substance alongside water. Nevertheless, monitoring this parameter remains essential for understanding the matrix behavior. Since all measurements were conducted under identical instrumental conditions, the potential minor loss of the API occurs uniformly across all samples, thereby ensuring a highly valid and rigorous comparative analysis between the different film formulations. A comparative analysis showed that the moisture content was significantly influenced by the structural features resulting from each forming method (two-way ANOVA, *p* < 0.05). Moreover, the post hoc analysis revealed that the introduction of menthol led to a statistically significant increase in moisture content across all fabrication techniques (Tukey’s test, *p* < 0.05). Those obtained by the electrospinning method showed the highest moisture content overall (14.38 ± 0.38% and 17.71 ± 0.48%, respectively), which correlates with their greater thickness and porous fiber network, providing a larger volume available for water retention. While those obtained by 3D printing showed the lowest hydration levels (12.58 ± 0.18% and 15.12 ± 0.22%), likely due to the highly compacted layer-by-layer structure achieved during extrusion, demonstrating a significant difference from the electrospun samples (*p* < 0.05). According to the literature, the moisture content of PVA-based films varies depending on their composition and the drying conditions. PVA films are highly hydrophilic due to the large number of hydroxyl groups that interact with water molecules. This makes moisture absorption an important characteristic of such materials, significantly affecting their properties when exposed to environmental humidity. PVA films obtained by the solvent casting method demonstrated a moisture content of up to approximately 21% in a high-humidity environment. However, modifying the composition resulted in changes to the moisture content, depending on the structure and interactions of the components within the film matrix. For instance, introducing stabilizers or fillers reduced or altered the material’s ability to absorb moisture, due to changes in the number of available hydrophilic groups and the degree of crystallinity of the matrix [88]. Mitrea et al. [89] demonstrated that the moisture content of PVA films can vary from approximately 3.75% to 26.91%, depending on the composition (e.g., up to ~26.9% with glycerine). Cautela et al. [90] found the moisture content of the films containing PVA and pectin to be from 5% to 8.7%. In addition, the moisture content of the commercially available Vaginal Contraceptive Film^®^ (VCF^®^) (Apothecus, Oyster Bay, NY, USA) was found to be 13.1% [87]. Thus, the moisture content observed in this study falls within the ranges reported in the literature, indicating an appropriate level of hydration that ensures the films have balanced mechanical and functional properties.

As is well known, the physicochemical properties of films, such as their microstructure, thickness and moisture content, play a crucial role in determining their textural characteristics, including their strength and elasticity [91]. Thus, the folding endurance tests showed that all the samples had good mechanical stability and elasticity, which ensures their integrity remains intact when they are bent repeatedly.

The bursting strength of blank films produced using all forming methods was 1.5–1.75 times greater than that of menthol-containing films, representing a statistically significant decrease upon incorporation of the additive (two-way ANOVA, *p* < 0.05). The decrease in this indicator following the introduction of menthol may be due to the weakening of interchain interactions in the PVA matrix, resulting in a partial disruption to its structural integrity. This is consistent with SEM analysis data indicating the formation of a porous microstructure. Similar behaviour has been observed in other studies involving pure PVA film [92].

At the same time, introducing menthol into the PVA film matrix led to a rise in tensile distance, indicating increased deformability of the material. The enhanced elongation is probably due to the plasticising effect of menthol and the films’ higher moisture content, which increases the mobility of the polymer chains. The films obtained by 3D printing method had the highest tensile distance values among the menthol-containing samples (10.62 ± 0.71 mm), showing a statistically significant difference compared to the other fabrication methods (Tukey’s test, *p* < 0.05), which may be due to the peculiarities of their microstructure, as revealed by SEM analysis, and the layer-by-layer nature of matrix formation.

A similar trend was also observed in tensile strength: in the presence of menthol, this parameter maintained stable values or showed a slight upward trend, although this directional shift was not statistically significant in all pairs (*p* > 0.05), indicating that resistance to tensile stress was preserved alongside increased film plasticity. This combination of parameters indicates that the material is transitioning to a state that is more ductile but less resistant to localized failure, which is consistent with a decrease in bursting strength. Analogous changes were observed in the work of Ojagh et al. [93], who demonstrated that introducing cinnamon essential oil, which exhibits plasticizer-like behaviour, results in significant alterations to the structure and mechanical properties of chitosan-based films, including an increase in tensile strength.

The adhesive properties of the films were evaluated on a pre-moistened surface, as minimal adhesion was observed for dry samples [94]. For films produced using solvent casting and 3D printing, surface wetting increased adhesiveness, which logically correlates with an improvement in the deformability and the ability to form close contact with the probe. Specifically, the introduction of menthol led to a statistically significant increase in adhesiveness for both the 3D-printed (F1) and solvent-casted (F2) formulations (Tukey’s test, *p* < 0.05), demonstrating that the presence of the plasticizer enhances the matrices’ capability to interact with the moistened substrate.

Films obtained by electrospinning exhibited different behaviour during adhesion testing. When the surface was moistened and subjected to stress in the area of contact with the probe for 60 s, significant thinning and partial dissolution of the material was observed, despite these films being thicker. This behaviour may be due to the fibrous microstructure revealed by SEM analysis, the PVA matrix’s high water sensitivity, and its rapid swelling upon contact with moisture. Consequently, it proved difficult to obtain reproducible and accurate values for the adhesiveness index of films formed by electrospinning.

**Figure 6:**
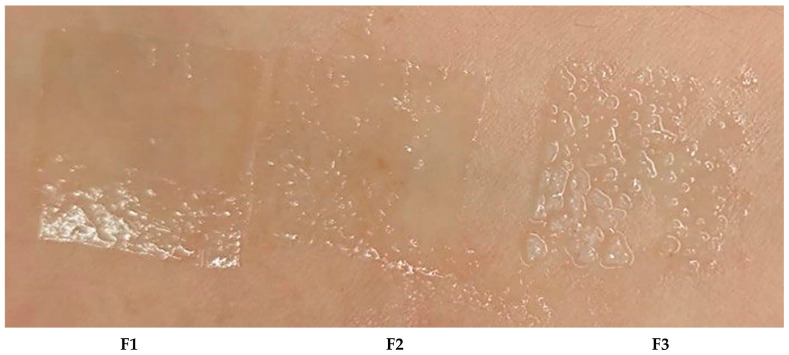
The behaviour of PVA films containing menthol, fabricated using different methods, when applied to moisturized skin on the hands: F1—3D printing; F2—solvent casting; F3—electrospinning.

This behaviour was confirmed through visual observation when applying the film to moisturized skin on the hand (Figure 6). Samples obtained using the electrospinning method (F3) demonstrated local softening and partial dissolution upon contact with moist skin. In contrast, samples formed using other methods retained their structural integrity to a greater extent. This suggests that the PVA matrix, created using the electrospinning method, is more sensitive to the effects of moisture. Guo Xiong with co-authors [95] and Zehra İrem Yıldız and co-author [19] also established that electrospun films disintegrate and dissolve very fast.

Overall, the obtained results demonstrate a relationship between the physicochemical and mechanical properties of the films and their internal structure. The degree of molecular organization and phase distribution of menthol within the PVA matrix were strongly influenced by the fabrication method, resulting in variations in polymer chain packing, free volume, and microstructural uniformity, as evidenced by SEM and XRD analyses. XRD confirmed a predominantly amorphous nature of all PVA-based systems while revealing differences in residual ordering and menthol crystallinity among formulations. These findings were consistent with SEM observations, which demonstrated method-dependent morphological features ranging from dense continuous films to more porous fibrous structures. Consequently, variations in film thickness, moisture content, and mechanical behaviour can be attributed to differences in matrix compaction and intermolecular interactions governed by the fabrication process. In general, less ordered and more porous structures (electrospun films) exhibited higher moisture content and increased deformability, whereas more compact morphologies (3D-printed and solvent-cast films) showed lower hydration and higher mechanical resistance.

**Figure 7:**
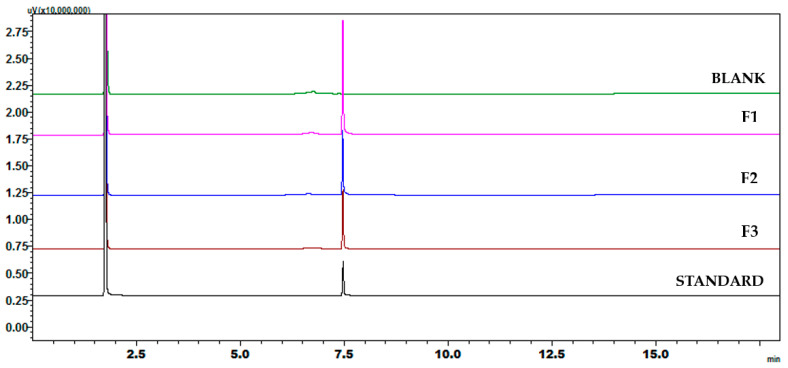
GC/FID chromatograms of PVA films with menthol fabricated by different methods: F1—3D printing, F2—solvent casting, F3—electrospinning; blank film and menthol standard included.

Determining the residual content of volatile components in films, particularly menthol, is key to assessing their stability and effectiveness in retaining them in the polymer matrix [96,97]. This analysis enables a quantitative comparison of the menthol content and distribution in films produced using different forming methods. It also allows the impact of production technology and drying conditions on the matrix’s ability to retain volatile APIs to be assessed. Figure 7 shows representative GC/FID chromatograms of the investigated film formulations together with a blank film and a menthol standard.

According to the obtained data, the method used to form the films significantly influenced the final active substance content and its entrapment efficiency (%EE) within the polymer matrix. The reported percentages represent the actual menthol content determined in the dried film matrices. Given that the volatile solvents (water and ethanol) evaporate during processing, a notable loss of menthol via co-evaporation was observed across all methods compared to the theoretical loading (27.78% in the dry residue). Specifically, films formed by the solvent casting method (F2) exhibited the lowest menthol content (3.34%; %EE = 12.02%). This can be explained by the initial solution spreading into a thin layer on the substrate, which increases the evaporation surface and causes partial menthol volatilization during drying. Using electrospinning (F3), the menthol content was intermediate (3.66%; %EE = 13.18%)—the layer was thicker than with solvent casting, but the total surface area was larger than with 3D printing, leading to partial loss of the API. Conversely, the 3D printing method (F1) allowed the highest amount of menthol to be retained (4.0%; %EE = 14.40%) by forming dense, extruded layers with a reduced open surface area, which effectively minimized the loss of the volatile component. It should be noted that 3D printing involved thermal drying at 35 °C, whereas films produced by electrospinning were dried at room temperature. Nevertheless, 3D printing resulted in greater menthol retention, reflecting its influence on the formation of a dense, compact polymer matrix structure that effectively locks the volatile API within its framework. Therefore, the fabrication method determines the internal structure of the matrix and consequently dictates the retention efficiency of volatile components, regardless of the drying temperature.

The observed entrapment efficiency values (12–15%) are entirely typical for open film matrix drying systems involving highly volatile monoterpenes. For instance, Sapper et al. evaluated polyvinyl alcohol (PVA) films plasticized with glycerol containing a volatile terpene (carvacrol) prepared via the solvent casting method [98]. The authors reported that despite drying at room temperature, a dramatic volatilization of the active component occurred, resulting in carvacrol retention values dropping significantly to approximately 10–13% in the dry films. This benchmark study confirms that the low retention rates achieved in our work comply with standard formulation behaviors for highly volatile substances embedded in hydrophilic polymer matrices during solvent evaporation.

**Figure 8:**
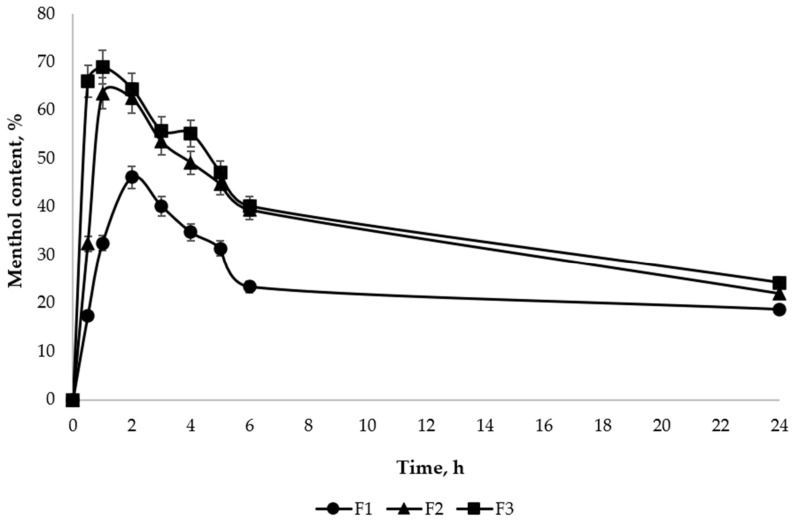
Time-dependent profiles of menthol content in the dissolution medium released from PVA-based films prepared by different fabrication methods: F1—3D printing, F2—solvent casting, F3—electrospinning (data represent mean ± SD, *n* = 3). Statistical significance was evaluated using one-way analysis of variance (ANOVA) followed by Tukey’s multiple comparisons post hoc test; differences between all three formulations (F1, F2, and F3) were found to be statistically significant (*p* < 0.05) at key release intervals up to 6 h.

Following the quantification of the initial API load in the films prepared by the three distinct methods and the visual confirmation of matrix disintegration upon contact with skin moisture (Figure 6), it was critical to evaluate how these structural differences influence menthol release upon exposure to a liquid medium. To this end, an in vitro release study was performed over 24 h. A water:ethanol mixture (70:30, *v/v*) was selected as the dissolution medium to balance the contrasting solubilities of the system components, promoting hydration and disintegration of the hydrophilic PVA matrix while ensuring sufficient solubilization of hydrophobic menthol. Because menthol is a highly volatile compound, the release data were not expressed as conventional cumulative release profiles. Classical cumulative release analysis assumes that the released drug remains quantitatively within the dissolution medium throughout the experiment [99,100,101]. In contrast, menthol continuously partitions into the vapor phase and may evaporate from the medium during prolonged testing. Therefore, the release behavior was evaluated using the experimentally detected amount of menthol present in the dissolution medium at each sampling time point, expressed as a percentage of the initial menthol content of the respective formulation (Figure 8).

All formulations exhibited a characteristic biphasic profile consisting of an initial increase in menthol concentration during the first 1–2 h, followed by a gradual decline up to 24 h. The initial phase reflects rapid matrix hydration and transfer of menthol into the surrounding medium. The subsequent decrease is consistent with progressive menthol volatilization from the dissolution system, which eventually exceeds the rate at which additional menthol is supplied from the matrix. Despite having identical qualitative compositions, the three fabrication methods produced markedly different release profiles, highlighting the critical role of matrix morphology in controlling menthol transport. Statistical analysis revealed that the differences in release kinetics between the three manufacturing techniques were significant (*p* < 0.05) across key time points.

The electrospun nanofibrous formulation (F3) demonstrated the fastest release kinetics, reaching a maximum detected value of 68.94 ± 3.45% of the initial menthol content after 1 h. This behavior can be attributed to the highly porous structure and exceptionally large specific surface area of the nanofiber network, which facilitates rapid medium penetration and immediate exposure of menthol-containing domains. As a result, a substantial fraction of the incorporated menthol became available in the dissolution medium within a short period of time.

The solvent-cast films (F2) exhibited intermediate behavior, reaching a peak value of 63.48 ± 3.17% after 1 h. Although the cast films also hydrated rapidly, their continuous planar morphology provided a lower surface area than the electrospun matrices, resulting in a less pronounced burst effect. Nevertheless, the relatively homogeneous structure still enabled efficient transfer of menthol into the medium during the early stages of dissolution. Notably, during the first hour of dissolution, the measured amount of menthol in the medium from F3 was statistically significantly higher compared to F2 (*p* < 0.05).

In contrast, the 3D-printed films (F1) showed the lowest and most delayed maximum value, reaching only 46.14 ± 2.31% after 2 h. This behavior is likely associated with the dense layer-by-layer structure generated during extrusion-based printing. Upon hydration, the outer regions of the printed strands become swollen and highly hydrated, while the inner regions remain comparatively less accessible to the dissolution medium. Consequently, the hydrated polymer layer may temporarily increase diffusional resistance and slow the transfer of menthol from the matrix into the surrounding medium. This interpretation is supported by the delayed concentration maximum observed for F1 compared with F2 and F3. Throughout the entire 24-h dissolution study, the detected menthol concentration in the medium for the 3D-printed films (F1) remained statistically significantly lower (*p* < 0.05) than that of both the solvent-cast (F2) and electrospun (F3) formulations, confirming a highly controlled and prolonged release matrix.

Overall, the results demonstrate that the fabrication method has a pronounced influence on menthol release behavior, with differences in polymer matrix structure resulting in distinct release profiles. Among the investigated formulations, the 3D-printed films provided the most sustained menthol release, whereas the electrospun and solvent-cast systems promoted faster release during the initial stages of the dissolution study.

From a formulation perspective, the sustained release profile observed for the 3D-printed matrices may be advantageous for topical and transdermal delivery of volatile compounds. By moderating menthol release, the printed structure appears to prolong the availability of the active ingredient compared with the solvent-cast and electrospun systems.

Due to menthol’s pharmacological activity and ability to penetrate the skin, a key part of the study was not only investigating its release behavior but also evaluating its transdermal permeation profile. To this end, the skin permeation fluxes of menthol into the epidermis and dermis layers were analyzed depending on the film fabrication method. Permeation studies were conducted over a 24-h period using Bronaugh-type vertical flow-through diffusion cells. This allowed for an accurate assessment of the influence of film formation technology on the transdermal delivery rate of menthol and its behavior in the ‘film-skin’ system. The resulting skin permeation fluxes from the different film formulations are presented in Table 5.

The ex vivo permeation studies demonstrated that the fabrication technology deeply influences the transdermal delivery profile of menthol. Quantitatively, the 3D-printed films (F1) provided a statistically significant increase in epidermal flux compared to the solvent-cast (F2) and electrospun (F3) matrices—surpassing them by 4.4 and 53.1 times, respectively (one-way ANOVA followed by Tukey’s post hoc test, *p* < 0.05). This facilitated the effective formation of a pronounced epidermal drug depot. Concurrently, the differences in dermal flux between the fabrication methods were less pronounced, maintaining comparable values across all formulations (*p* > 0.05), with F1 showing a modest increase of 1.26 and 1.34 times over F2 and F3, respectively.

These observed permeation dynamics strongly correlate with the matrix microstructure, dissolution pathways, and the behavior of volatile component in an open “film–skin” system. Upon contact with the hydrated skin surface, the tested formulations exhibited drastically different structural transformations. The fibrous, highly porous nanostructure of the electrospun films (F3) provides an extremely large specific surface area, which triggers an immediate burst release, rapidly liberating 68.94 ± 3.45% of the menthol within the first hour. This explosive matrix disintegration causes the highly volatile API to prematurely evaporate into the ambient air before it can effectively partition into the lipophilic stratum corneum. This severe loss of volatile component directly explains the critically low epidermal flux (0.027 ± 0.001 μg/cm^2^/h) and compromised skin accumulation observed for F3. A similar, though less extreme, phenomenon occurs with the solvent-cast films (F2), where rapid initial dissolution (63.48 ± 3.17% release at 1 h) and subsequent premature volatilization limited its transdermal delivery efficiency.

Conversely, the dense, layer-by-layer polymeric framework fabricated by 3D printing (F1) successfully restricts rapid, moisture-induced dissolution. By minimizing the premature loss of the volatile compound during the initial phase (releasing only 32.49 ± 1.62% at 1 h), the printed matrix ensures a stable, controlled diffusion pathway and prolonged contact with the skin surface. This structural protection maintains a sustained concentration gradient across the skin barrier over the 24-h period, successfully maximizing transdermal fluxes and driving the targeted accumulation of menthol within the skin layers.

Ultimately, the collective findings confirm that the 3D printing method provides distinct advantages over solvent casting and electrospinning. The matrix design achieved through printing plays a central role in modulating both the dissolution rate and subsequent transdermal fluxes, demonstrating how manufacturing technology can optimize the delivery performance of volatile compounds within polymeric matrices.

Furthermore, the stability of the developed polymeric films after 6 months of storage was evaluated in terms of physical appearance and menthol content retention. No visible changes in colour, transparency, flexibility, or surface integrity were observed in any formulation, indicating good physical stability under the applied storage conditions. In contrast, the primary changes during storage were associated with menthol loss, which strongly depended on the fabrication method. After 6 months, the 3D-printed films (F1) retained a menthol content of 3.0% (representing 75.0% retention of the initial content determined immediately after preparation), whereas the solvent-cast (F2) and electrospun (F3) films retained 1.19% (35.6% retention) and 1.02% (27.9% retention), respectively. These results confirm that the choice of manufacturing technology plays a critical role in governing the long-term preservation of volatile active compounds within solid polymeric matrices.

In the literature, it has been demonstrated that the stability of volatile compounds in polymeric films is primarily controlled by solvent evaporation behaviour and the resulting internal morphology, which determines mass transport pathways within the matrix [102,103]. In solvent-cast films, slow solvent removal may induce phase separation and the formation of heterogeneous structures with more developed pore networks, which facilitate diffusion and loss of menthol during storage. Electrospun systems, in contrast, exhibit highly porous fibrous morphologies with large surface areas, which may further enhance volatilization unless stabilizing interactions are present [104].

These microstructural differences were confirmed by SEM analysis in the present study. 3D-printed films exhibited a compact structure with only superficial surface irregularities and shallow microindentations, whereas solvent-cast and electrospun systems showed more open and interconnected morphologies. This structural compactness in 3D-printed films limits internal diffusion pathways, thereby minimizing menthol loss during storage, whereas the more porous matrices of the alternative formulations exhibit a more pronounced reduction in active substance content.

Therefore, consistent with the findings obtained immediately after preparation, 3D-printed matrices exhibited the highest capacity for menthol retention, which was maintained after storage. Importantly, since menthol loss during storage could significantly compromise the therapeutic performance and bioavailability of the final formulation, maximizing volatile agent entrapment is critical to ensuring consistent clinical efficacy. However, it should be noted that longer-term stability studies, including the assessment of additional physicochemical and performance characteristics, are still ongoing and will be necessary to fully establish the storage stability of the developed films.

## 4. Conclusions

For the first time, a comprehensive comparative study of menthol-containing PVA-based transdermal films fabricated by 3D printing, solvent casting, and electro-spinning was conducted. Both the fabrication method and menthol acted as structure-modifying factors governing the internal organization of the polymer matrix, thereby determining the resulting microstructure and, consequently, the physicochemical and textural properties of the films as well as the release, permeation, and stability behaviour of the volatile component.

The fabrication method defined distinct microstructural characteristics: 3D printing generated a compact, ordered, and layered structure with limited porosity, solvent casting produced dense continuous films with a smooth morphology, and electrospinning yielded a highly porous fibrous network with extensive inter-fibre voids. Menthol further modified the PVA matrix by disrupting intermolecular interactions and increasing polymer chain mobility, leading to changes in film organization and structural integrity. As a result, electrospun and solvent-cast films exhibited higher moisture content, lower mechanical strength, and greater deformability, as well as reduced menthol retention, compared with the more compact and ordered structure of 3D-printed films.

Menthol losses were governed by microstructure-dependent pathways and occurred during processing, dissolution, and storage. Electrospun and solvent-cast films exhibited greater menthol losses, whereas 3D-printed films demonstrated higher retention and stability across all stages.

The release profiles of menthol were in agreement with ex vivo skin permeation data, confirming a direct relationship between microstructure, release kinetics, and transdermal transport. Rapid release and faster depletion observed in electrospun and solvent-cast films were associated with more open or less compact structures, whereas the layered polymer matrix of 3D-printed films enabled sustained and more controlled delivery, maintaining prolonged menthol availability for skin diffusion and resulting in higher dermal fluxes.

Overall, the results highlight 3D printing as a particularly promising fabrication strategy for transdermal delivery systems containing volatile active compounds, as its precise layer-by-layer construction enables effective control over microstructure, thereby minimizing premature volatilization and ensuring sustained and predictable release and transport behaviour.

Despite the comprehensive analysis, several limitations should be acknowledged. The ex vivo skin model does not fully replicate in vivo physiological conditions, including dynamic blood perfusion, metabolic activity, and barrier renewal processes, which may influence transdermal transport and long-term retention. In addition, intradermal distribution of menthol was not resolved in a depth-dependent manner; therefore, a detailed layer-specific penetration profile remains to be established. Furthermore, longer-term stability studies, including comprehensive physicochemical and performance evaluations, are required to assess the storage behaviour of the developed films.

## Figures and Tables

**Table 1:** Film formulation compositions.

Sample	Ingredients, % *w*/*w*					Fabrication Method
	Menthol	PVA	Glycerol	Ethanol	Water Purified	
F1	5.0	10.0	3.0	40.0	42.0	3D printing
F2	5.0	10.0	3.0	40.0	42.0	Solvent casting
F3	5.0	10.0	3.0	40.0	42.0	Electrospinning

**Table 2:** Optimization of water–ethanol solvent ratios for PVA/menthol film-forming solutions.

Ethanol to Water Ratio (*w*/*w*)	Polymer Behaviour (10% PVA)	API Behaviour (5% Menthol)	Visual and Technological Outcome
0.5:1	Complete dissolution with no visible undissolved material.	Partial solubilization; visible undissolved material remained.	Turbid, opalescent system with visible phase separation and coalescence of menthol droplets at the surface.
1:1	Full solubility; homogeneous solution with no visible signs of aggregation or precipitation.	Complete solubilization with no visible undissolved material.	Homogeneous, single-phase, stable opalescent solution with no evidence of phase separation.
1.5:1	Rapid polymer precipitation, resulting in a heterogeneous system with visible solid aggregates.	Rapid and complete solubilization.	Visible polymer aggregation and heavy precipitation (white flakes); a heterogeneous multiphase system with complete loss of homogeneity.

Note: All formulations were evaluated at 25 °C under continuous magnetic stirring before visual inspection.

**Table 3:** Effect of PVA concentration on rheology and processability of film-forming formulations.

PVA Concentration (*w*/*w*)	Rheological Status/Dynamic Viscosity	Technological Outcome by Processing Method		
		SSE 3D Printing	Solvent Casting	Electrospinning
5%	Semi-dilute unentangled regime (near-Newtonian, low elasticity)/32.41 ± 1.15 mPa·s	Rapid spreading of deposited material with immediate track widening, resulting in loss of shape fidelity and structural collapse; no continuous film formation achieved.	Rapid spreading with fast deaeration, yielding very thin films with limited structural integrity that are fragile, prone to rupture due to low thickness and strong substrate adhesion.	Continuous jet disruption due to capillary instability, leading to a transition from electrospinning to electrospraying with bead/droplet deposition, resulting in non-uniform and low-quality nanofibrous mats.
10%	Semi-dilute entangled regime (viscoelastic, shear-thinning behavior)/159.03 ± 0.72 mPa·s	Stable extrusion with good shape retention and preserved layer geometry; continuous and uniform film formation achieved upon deposition.	Homogeneous spreading with controlled evaporation, yielding high-quality films with preserved geometry and easy detachment from the substrate.	Stable, continuous jet formation, yielding uniform, dry, high-quality nanofibrous mats with good structural consistency.
15%	Concentrated regime (high-viscosity, gel-like network)/745.20 ± 8.63 mPa·s	High flow resistance requiring increased extrusion pressure, with risk of premature drying and nozzle clogging; non-uniform and discontinuous film formation observed.	Air bubble entrapment during casting, leading to non-uniform film formation and reduced film quality, with shrinkage and distortion of the dried matrix.	Restricted mass transfer and rapid polymer solidification at the needle tip, causing capillary clogging and process interruption, resulting in incomplete and defective nanofibrous mat formation.

**Table 4:** The physicochemical and textural characteristics of PVA-based film formulations fabricated using different methods (*n* = 5; data represent mean ± SD. Statistical significance was determined using two-way ANOVA followed by Tukey’s post hoc test; *p* < 0.05).

Parameters	Samples/Results					
	F1 (3D Printing Method)		F2 (Solvent Casting Method)		F3 (Electrospinning Method)	
	Without Menthol	With Menthol	Without Menthol	With Menthol	Without Menthol	With Menthol
Thickness, μm	167 ± 13	174 ± 14	155 ± 9	171 ± 15	180 ± 12	203 ± 18
Moisture content, %	12.58 ± 0.18	15.12 ± 0.22	12.98 ± 0.07	16.24 ± 0.31	14.38 ± 0.38	17.71 ± 0.48
Folding endurance	>300	>300	>300	>300	>300	>300
Bursting strength, N	23.11 ± 0.92	13.25 ± 0.65	22.41 ± 1.48	13.94 ± 1.12	21.22 ± 1.39	14.18 ± 0.22
Tensile distance, mm	4.98 ± 0.19	10.62 ± 0.71	6.29 ± 1.75	8.65 ± 1.09	5.58 ± 0.62	5.91 ± 0.51
Tensile strength, N/mm	1.685 ± 0.049	1.761 ± 0.328	1.244 ± 0.098	1.315 ± 0.169	1.275 ± 0.131	1.492 ± 0.174
Adhesiveness, N	3.20 ± 0.33	5.15 ± 0.27	3.35 ± 0.28	5.76 ± 0.54	–	–

Note: Folding endurance is reported as >300, indicating that all tested film formulations withstood 300 folding cycles without any signs of cracking or breaking, at which point the test was intentionally terminated according to the protocol; “–”—adhesion characteristics were not determined due to film failure during testing.

**Table 5:** Epidermal and dermal permeation fluxes of menthol from PVA-based films depending on the fabrication method (*n* = 3; data represent mean ± SD; evaluated via one-way ANOVA followed by Tukey’s post hoc test).

Parameters	Samples/Results		
	F1 (3D-Printing Method)	F2 (Solvent Casting Method)	F3 (Electrospinning Method)
Epidermis flux (μg/cm^2^/h)	1.433 ± 0.246 ^a^	0.325 ± 0.054 ^b^	0.027 ± 0.001 ^c^
Dermis flux (μg/cm^2^/h)	3.983 ± 0.208 ^d^	3.171 ± 0.333 ^d^	2.979 ± 0.588 ^d^

Note: Different superscript letters (^a,b,c,d^) within the same row indicate statistically significant differences between the film formulations (*p* < 0.05, Tukey’s post hoc test). Values sharing the same superscript letter are not statistically significant (*p* > 0.05).

## Data Availability

The original contributions presented in the study are included in the article, further inquiries can be directed to the corresponding author.
